# A tangible and interactive viral model for enhancing virology education: using hepatitis B virus as a model system

**DOI:** 10.3389/fmicb.2026.1825214

**Published:** 2026-07-01

**Authors:** Jincheng Li, Leiliang Zhang

**Affiliations:** 1 Department of Clinical Laboratory Medicine, The First Affiliated Hospital of Shandong First Medical University & Shandong Provincial Qianfoshan Hospital, Jinan, Shandong, China; 2 Department of Pathogen Biology, School of Clinical and Basic Medical Sciences, Shandong First Medical University & Shandong Academy of Medical Sciences, Jinan, Shandong, China

**Keywords:** ELISA, hepatitis B panel, hepatitis B virus, interactive teaching model, microbiology education

## Abstract

Hepatitis B virus (HBV) infections continue to pose a significant global health challenge each year, highlighting the critical need for a comprehensive understanding of this hepatotropic DNA virus within the context of virology education. Many undergraduate students, however, struggle to master the complex architecture of HBV, particularly the three-dimensional structure of the Dane particle and the immunological principles underlying the Hepatitis B Panel, when relying solely on traditional two-dimensional representations. To address this educational gap, we developed a tangible, interactive, and cost-effective model using 3D-printed components, transparent acrylic, and magnetic elements. Key features of the model include a magnet-enhanced design that replicates the specific binding logic of Sandwich and Competitive ELISA, a bisected capsid that reveals internal genomic material, and a dual-purpose base that simulates host receptor to illustrate viral entry and therapeutic interventions. By transforming molecular interactions into a tactile experience, this cost-effective and biohazard-free model enhances student comprehension of HBV pathogenesis and clinical diagnostics, thereby supporting global efforts to expand hepatitis testing and education.

## 1 Introduction

Hepatitis B virus (HBV) is an enveloped, hepatotropic DNA virus that continues to pose a significant global health burden (Danpanichkul et al., 2025). Despite the availability of effective vaccines (Michel et al., 2011) and antiviral therapies (Menéndez-Arias et al., 2014), their impact is often limited by a persistent reservoir of undiagnosed infections and inadequate public awareness, both of which hinder global efforts to eliminate HBV (Hsu et al., 2023). For undergraduate students, a thorough understanding of HBV’s complex structural biology (Venkatakrishnan and Zlotnick, 2016) and the principles underlying its clinical detection (Liu et al., 2024) is essential for effective patient care and for advancing virological research. However, mastering these concepts remains a considerable educational challenge.

HBV exhibits a unique and intricate structure (Dryden et al., 2006). The infectious Dane particle comprises an outer envelope displaying three forms of hepatitis B surface antigen (HBsAg), designated large (L), middle (M), and small (S), which surround an icosahedral nucleocapsid composed of hepatitis B core antigen (HBcAg) (Venkatakrishnan and Zlotnick, 2016). Within this capsid resides the viral genome: a partially double-stranded relaxed circular DNA (rcDNA) (Summers et al., 1975) maintained in a circular conformation by cohesive sticky ends, along with the HBV polymerase, which contains three functional domains including terminal protein (TP), reverse transcriptase (RT), and RNase H (Venkatakrishnan and Zlotnick, 2016). These structural features directly inform clinical diagnostics, as the Hepatitis B Panel detects specific viral antigens (HBsAg, HBeAg, HBcAg) and their corresponding antibodies to diagnose infection stage and guide treatment decisions (Jakob, 1995). Yet, for many students, the three-dimensional architecture of the Dane particle and the dynamic molecular interactions governing these serological markers remain abstract and difficult to visualize.

Traditional instructional approaches, largely reliant on static two-dimensional diagrams and conventional Enzyme-Linked Immunosorbent Assay (ELISA) laboratory exercises, often fail to convey these complex spatial relationships. While innovative technologies such as virtual reality simulations have shown promise in enhancing engagement and comprehension, their widespread adoption is frequently constrained by high costs and technological infrastructure requirements (Tsai et al., 2023; Chitra et al., 2024). Hands-on teaching tools have emerged as effective alternatives in microbiology education (de Souza et al., 2020; Harris et al., 2022; Moreno et al., 2023; Zaaijer and Groen, 2025). For instance, tactile teaching tools with guided inquiry learning (TTT-GIL) have been successfully employed to teach antibody-epitope interactions and MHC haplotype concepts, demonstrating significant learning gains across diverse student populations (Harris et al., 2022). Such approaches align with constructivist learning theory (Gao et al., 2025; Yang et al., 2025), which posits that learners actively construct knowledge through experience, and embodied cognition principles, suggesting that physical interaction with learning materials enhances conceptual understanding and retention. Herman et al. demonstrated that tactile models of proteins, created through rapid prototyping, significantly enhance student engagement and comprehension, particularly among learners with lower visuospatial abilities (Herman et al., 2006). They proposed a “concreteness fading” strategy, which involves introducing concrete physical models before transitioning to abstract computer visualizations. This strategy has been incorporated into the design of our HBV model. With advancements in 3D printing, researchers have developed detailed protocols for fabricating complex macromolecular assemblies, such as HIV capsids and ribosomes, allowing for exploration of structure-function relationships through tactile interaction (Da Veiga Beltrame et al., 2017). More recently, Guenther et al. found that involving undergraduate students in 3D printing projects within anatomy and molecular biology courses not only increased their confidence with technology but also improved learning outcomes, with these benefits observed across all student groups (Guenther et al., 2021).

To address the need for accessible and pedagogically effective tools in virology education, we developed a low-cost, tangible, and interactive model of HBV. Constructed from 3D-printed components, transparent acrylic, and embedded magnets, this model visualizes the complete structure of the infectious Dane particle, including its envelope antigens (HBsAg L, M, and S), icosahedral nucleocapsid, and rcDNA genome with its associated polymerase. Critically, the strategic placement of magnets allows the model to simulate the specific binding logic underlying both sandwich and competitive ELISA formats, thereby clarifying the immunological principles of the Hepatitis B Panel. By transforming abstract molecular interactions into a visible, hands-on experience, this model engages multiple sensory modalities to deepen students’ comprehension of HBV pathogenesis, replication dynamics, and clinical diagnostics. Furthermore, experiential learning must integrate “hands-on” manipulation with “minds-on” reflection to achieve deep conceptual understanding (Mark, 2002). Our model is specifically designed to fulfill this dual requirement by combining the physical manipulation of viral components with structured reflective activities, such as ELISA simulations and schematic diagram analyses.

Beyond its educational value, the model is entirely biohazard-free, eliminating risks associated with handling infectious materials or toxic reagents in the teaching laboratory. This tangible learning tool offers undergraduate students an accessible, engaging, and memorable approach to mastering HBV, supporting broader efforts to enhance virology literacy and ultimately scale up hepatitis testing and education worldwide. Although this study focuses on HBV, the underlying design principles are broadly applicable to other viruses. This model serves as a proof of concept for a generalized approach to teaching viral structure and immunology through tangible, interactive models.

## 2 Materials and methods

### 2.1 Methods model design and structure

**FIGURE 1:**
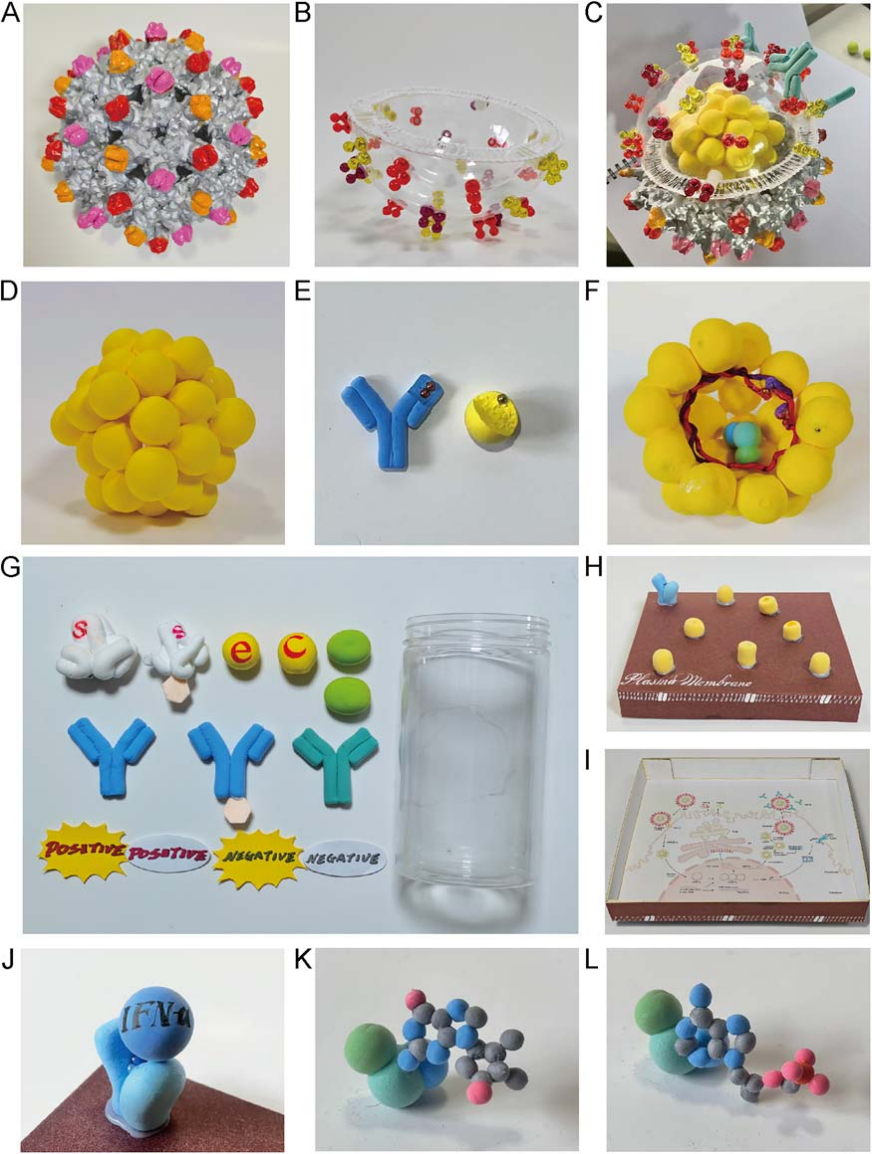
Components and assembly of the HBV model. **(A)** 3D-printed hemisphere representing the viral envelope. **(B,C)** Transparent acrylic hemisphere that allows for visualization of internal structures. **(D)** Icosahedral nucleocapsid constructed from lightweight clay. **(E)** Bisected capsid and antibody component, illustrating the placement of internal neodymium magnets. **(F)** Relaxed circular DNA (rcDNA) and HBV polymerase, with the terminal protein (TP), reverse transcriptase (RT), and RNase H domains depicted in green, cyan, and blue, respectively. **(G)** Upper surface of the base representing the host hepatocyte membrane. **(H)** Underside of the base featuring schematic diagrams. **(I)** ELISA simulation kit comprising transparent plastic jars, HBV antigens (HBsAg, HBeAg, HBcAg), antibodies, enzyme-labeled components, and four result stickers. The green antibody represents host-produced antibodies, while the pink hexagon denotes the enzyme label. **(J)** Interaction of IFN-α with IFNAR receptors. **(K)** Entecavir blocking the viral DNA polymerase. **(L)** Tenofovir inhibiting the viral DNA polymerase.

The model was designed to represent the complete structure of the Dane particle, which denotes the fully infectious HBV virion. The envelope consists of two hemispheres that are joined together. One hemisphere was 3D-printed to replicate the structure of the envelope proteins (Figure 1A), while the other hemisphere, constructed from transparent acrylic (Xinyuan Acrylic Products Factory) with an outer diameter of 20 cm, provides a clear view of the internal components (Figure 1B). To simulate the phospholipid bilayer, a smaller hemisphere with a diameter of 15.5 cm is nested concentrically inside a larger hemisphere. Short spacers of 2.25 cm are placed between the two hemispheres to maintain a consistent inter-layer gap.

### 2.2 Surface antigen representation

Magnetic pushpins (from Shenzhen Tiansheng Magnet Co., Ltd.), each 1.7 cm in length, were attached in pairs to the exterior surface to represent the surface antigen (HBsAg) dimers, which can interact with antibody components via magnets (Figure 1C). The red, pink, and orange pushpins symbolize the L, M, and S antigens, respectively.

### 2.3 Internal nucleocapsid construction

The internal nucleocapsid, characterized by its icosahedral structure, was constructed from super-light clay (Linyi Yilanzi Stationery Co., Ltd.), a type of paper clay (Figure 1D). Individual spherical core protein subunits were sculpted to a diameter of 3 cm. To allow visualization of the internal genetic material, the capsid was split into two halves. Neodymium beads (5 mm in diameter; from Pan’an County Leilei Buck Ball Toys Co., Ltd.) were embedded along the docking edges of the two halves (Figure 1E). Inside, the genetic material (the unique rcDNA) is represented by a dyed two-strand rope. Specifically, the genomic model consists of a red rope (length: 22 cm) with two magnetic beads attached at positions 0 cm and 19 cm from one end, and a purple rope (length: 19 cm) with two magnetic beads attached at 0 cm and 3 cm from one end. These precisely positioned beads mimic sticky ends, allowing the strands to physically adhere to each other and form a circular structure. The HBV polymerase, featuring three functional domains (TP, RT, and RNase H) was sculpted from super-light clay and placed within the capsid (Figure 1F).

### 2.4 ELISA simulation kit development

To familiarize students with the ELISA and the Hepatitis B Panel, we developed an ELISA simulation kit, scaling up each ELISA microplate well to a plastic jar (Yourun Plastic Packaging Factory Store) of 8.2 cm diameter and 15 cm height. This kit includes viral antigens (HBsAg, HBeAg, HBcAg), along with other solutes, corresponding antibodies, enzyme-labeled antigens and antibodies, and test result stickers (Figure 1G). Each component is equipped with predefined magnets to simulate protein-protein interactions.

### 2.5 Dual-purpose base

The model can be placed on a dual-purpose base. The top surface represents the membrane of the target cell, primarily the host hepatocyte, and features integrated NTCP receptors (Yan et al., 2012; Ni et al., 2014) and IFNAR. This configuration facilitates the modeling of viral infection processes and the resultant immune response effects (Figure 1H). On the underside of the base, there is a mechanism diagram containing schematics that illustrate the HBV replication cycle and various preventive and therapeutic mechanisms (Figure 1I). Additional components, such as IFN-α, entecavir, and tenofovir, were also created from super-light clay and incorporated with neodymium beads (Figures 1J–L).

## 3 Results

### 3.1 Visualizing viral architecture

**FIGURE 2:**
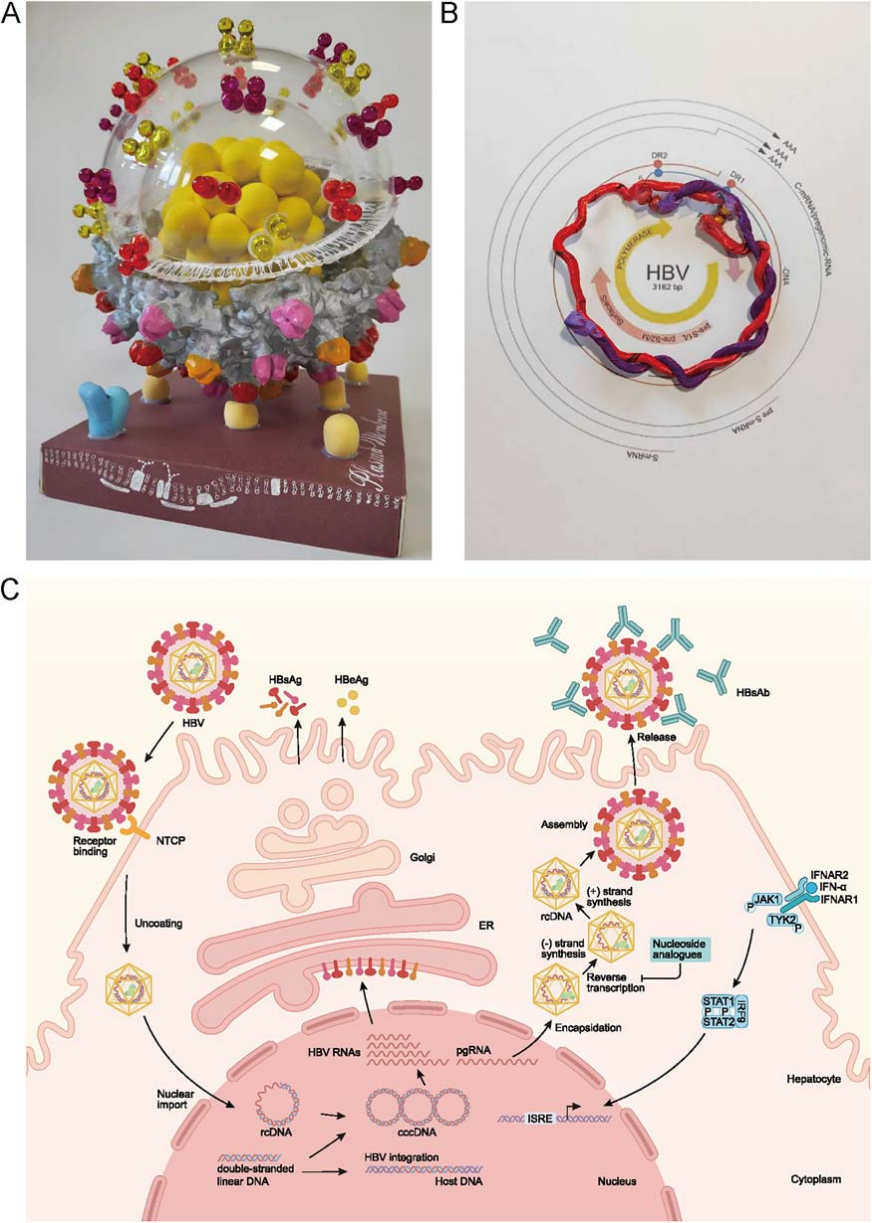
Structural details and virus-host interactions. **(A)** Envelope antigens docking onto the NTCP receptor on the simulated hepatocyte membrane. **(B)** HBV genome structure and organization, illustrating the partially double-stranded DNA with sticky ends. **(C)** Schematic diagram of the HBV life cycle, highlighting the inhibitory mechanisms of interferons, neutralizing antibodies, and antiviral agents.

The model provides a tangible, three dimensional representation of Hepatitis B virus and serves as an intuitive educational tool for understanding viral structure along with the mechanisms of infection, diagnosis, prevention, and treatment. It highlights detailed structural features and cross sectional views of the Dane particle. Students can easily observe the L, M, and S surface antigens displayed on the viral envelope (Figure 2A). The transparent acrylic hemisphere offers a clear view of internal viral components, including the nucleocapsid and encapsulated genetic material. The nucleocapsid can be separated into two halves to expose the viral genome and associated HBV polymerases. To illustrate the characteristic partial double stranded DNA structure, a rope constructed from two distinct strands coiled together effectively depicts the sticky ends that maintain its circular conformation (Figure 2B). In addition, the HBV polymerase is represented with its three functional domains: TP, RT, and RNase H.

### 3.2 Simulating viral entry and life cycle

The HBsAg proteins on the viral surface can be physically docked onto NTCP receptor sites located on the simulated hepatocyte membrane (Figure 2A). A colorful schematic diagram integrated into the model base provides an overview of the HBV life cycle and highlights the inhibitory mechanisms of interferons (Ashuo et al., 2025), neutralizing antibodies (Hong et al., 2019), and antiviral agents (Menéndez-Arias et al., 2014; Figure 2C).

### 3.3 Demonstrating ELISA detection principles

**FIGURE 3:**
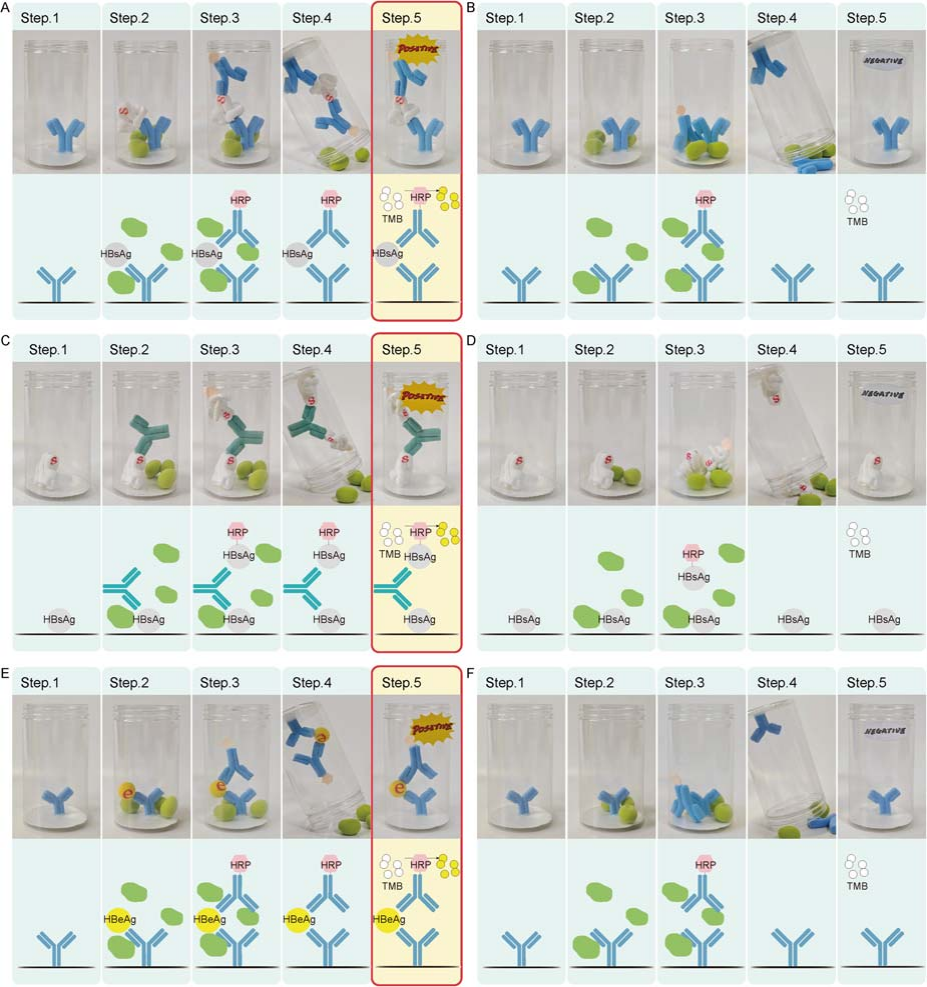
Simulation of sandwich ELISA for HBsAg, HBsAb, and HBeAg detection. **(A,B)** Step-by-step simulation of HBsAg detection in positive **(A)** and negative **(B)** samples. **(C,D)** Simulation of HBsAb detection in positive **(C)** and negative **(D)** samples. **(E,F)** Simulation of HBeAg detection in positive **(E)** and negative **(F)** samples.

To enhance familiarity with ELISA protocols and the clinical interpretation of HBV markers, the model simulates the detection principles that occur in standard, sample, and blank wells of an ELISA plate. For HBsAg detection, a coated antibody with specifically oriented magnetic poles in its antigen binding regions is adhered to the bottom of the model jar (Figure 3A, Step 1). When a positive sample is added and the jar is gently shaken, internal components come into proximity and allow specific binding via magnetic attraction, thereby mimicking epitope recognition (Figure 3A, Step 2). The subsequent addition of enzyme labeled antibodies completes the coated antibody–HBsAg–enzyme sandwich complex (Figure 3A, Step 3). Inverting the jar causes unbound components to fall out under gravity, simulating the washing step that removes unbound reactants (Figure 3A, Step 4). In a positive test, the enzyme-labeled antibodies remain securely in the jar, and a positive result is indicated by a sticker that adheres to the jar (Figure 3A, Step 5). In contrast, when a negative sample lacks HBsAg, no bridging antigen is available. The enzyme-labeled antibodies fail to bind and are discarded during washing, yielding a negative result (Figure 3B). This sandwich logic applies similarly to HBsAb and HBeAg detection (Figures 3C–F), where the target analyte (HBsAb or HBeAg) mediates magnetic attachment between the coated antibody and enzyme labeled antibodies.

### 3.4 Illustrating competitive ELISA

**FIGURE 4:**
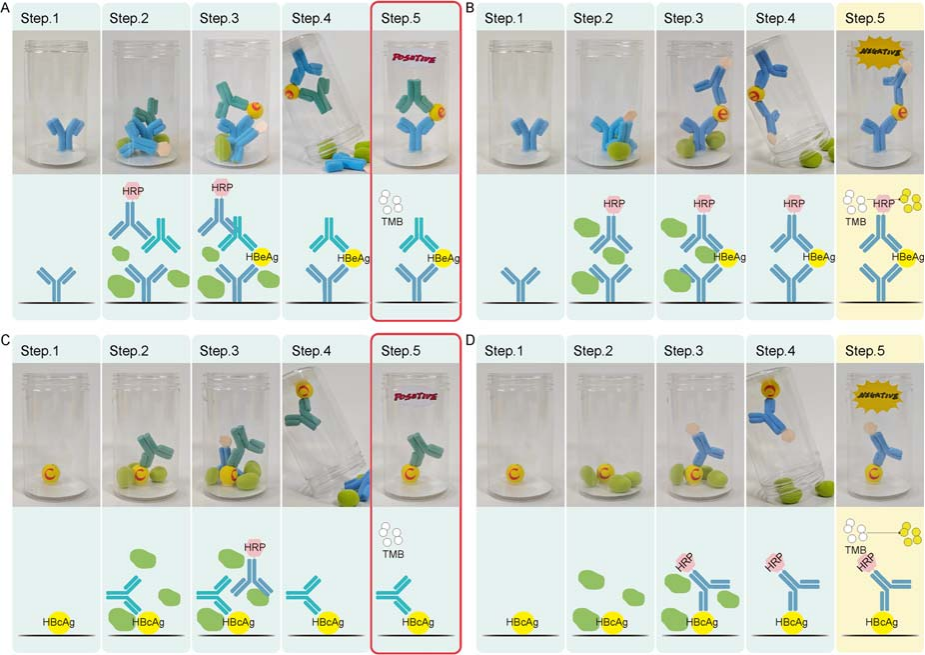
Simulation of competitive ELISA for HBeAb and HBcAb detection. **(A, B)** Simulation of HBeAb detection in positive **(A)** and negative **(B)** samples. **(C, D)** Simulation of HBcAb detection in positive **(C)** and negative **(D)** samples.

By contrast, the model effectively demonstrates the distinct logic underlying competitive ELISA for HBeAb and HBcAb detection. For HBeAb, the model jar is first coated with HBeAb (Figure 4A, Step 1). A positive sample, enzyme labeled HBeAb, and neutralizing antigen HBeAg are then added (Figure 4A, Steps 2–3). The sample antibody competes with the enzyme labeled antibody for binding to the neutralizing antigen, which is attached to the coated HBeAb. As a result, the enzyme labeled HBeAb fails to establish magnetic attachment to the jar bottom and is discarded during washing (Figure 4A, Step 4), leading to a positive sticker (Figure 4A, Step 5). In a negative case where the sample lacks HBeAb, no competition occurs. The enzyme labeled HBeAb binds to the jar bottom via the pre coated HBeAb and neutralizing antigen HBeAg, resulting in a negative test result (Figure 4B).

For HBcAb detection, the HBcAg component is pre adhered to the bottom of the model jar (Figure 4C, Step 1). Students then add the sample and enzyme labeled HBcAb. If the sample is positive, the sample antibodies compete for limited magnetic binding sites and prevent the enzyme labeled antibodies from anchoring (Figure 4C, Steps 2–3). Inverting the jar reveals the absence of bound enzyme, which indicates a positive result (Figure 4C, Steps 4–5). In a negative sample, enzyme labeled HBcAb binds directly to HBcAg without interference and remains in the jar after washing, representing a negative result (Figure 4D). By simulating the entire workflow from sample incubation to the final enzymatic reaction, this model transforms abstract antigen antibody detection principles into a visible, hands on learning experience.

### 3.5 Modeling therapeutic interventions

Finally, the physical model, together with its schematic diagram, illustrates the molecular mechanisms underlying common clinical interventions such as interferon, entecavir, and antibody therapies. For interferon therapy, the IFN-α component can be magnetically attached to IFNAR1/2 receptors on the simulated hepatocyte membrane base (Figure 1J). The accompanying signaling pathway diagram explains how IFN-α binding activates the JAK-STAT pathway and triggers the expression of interferon stimulated genes (Darnell et al., 1994; Figure 2C). For antiviral agents, structural analogues including entecavir and tenofovir attach to the viral DNA polymerase (Darnell et al., 1994; Figures 1K,L). This demonstrates how these nucleotide analogues inhibit HBV replication by physically blocking the polymerase catalytic site and halting viral DNA synthesis.

Vaccination and administration of human hepatitis B immunoglobulin (HBIG) stimulate the production of protective, high titer HBsAb antibodies in vivo (Hilleman, 1987). By using magnetic attraction between HBsAb components and HBsAg proteins on the surface of the virion model, the system shows how neutralizing antibodies block viral HBsAg and prevent viral entry into hepatocytes (Figure 1C). This antibody mediated protection is essential for hepatitis B vaccination, post exposure prophylaxis, and prevention of mother to child transmission.

### 3.6 Preliminary evaluation of learning outcomes

A preliminary evaluation involving 15 undergraduate students indicated that the model significantly enhanced self-reported understanding of HBV structure (mean score: 4.6/5) and ELISA principles (mean score: 4.4/5). Student feedback included comments such as: “I finally understand how sandwich ELISA works,” “The magnetic binding made the concept click for me,” and “Being able to physically separate the capsid helped me understand how the viral genome is packaged.” One student remarked, “I always confused HBeAg and HBcAg before, but seeing them as separate components in the model clarified the difference.” Constructive suggestions included, “Adding color-coded instructions for the ELISA steps would make it easier to follow,” and “It would be helpful to have more magnets on the antibodies to illustrate multiple binding sites.”

In addition to self-reported understanding, a preliminary knowledge assessment using five multiple-choice questions showed an improvement in average scores, rising from 62% before model use to 84% after model use (*n* = 15). Teacher observations during the laboratory session revealed that students actively engaged with both the model and each other. Groups were seen discussing the steps of sandwich ELISA while manipulating the components. One student explained to a peer, “See, the antigen has to be there to connect the two antibodies; that’s why the negative sample doesn’t stick.” Another group compared the sandwich and competitive ELISA formats, using the model jars to demonstrate the differences to one another. Students also spontaneously posed questions such as, “If the sample has both HBeAg and HBeAb, which one takes precedence?” and “Could this magnetic system be used to model other diagnostic tests like rapid antigen tests?” These questions stimulated further discussion and peer teaching within groups.

## 4 Discussion

### 4.1 Theoretical implications for virology education

The observed improvement in student comprehension can be interpreted through the lens of cognitive load theory (Szulewski et al., 2021; Zou et al., 2025). By externalizing complex three-dimensional relationships, the model reduces extraneous cognitive load, allowing students to focus on deeper conceptual understanding. By allowing students to physically manipulate viral components, the model transforms abstract concepts into concrete operations, thereby freeing cognitive resources for deeper processing. This interpretation is consistent with student comments indicating that “the magnetic binding made the concept click,” suggesting a moment of successful conceptual integration.

The model’s design also aligns with constructivist learning theory, which posits that learners actively construct knowledge through experience, and embodied cognition principles, which suggest that physical interaction with learning materials enhances conceptual understanding and retention. The hands-on nature of the model engages multiple sensory modalities, potentially creating stronger memory traces compared to passive learning through diagrams or lectures alone.

### 4.2 Pedagogical value in teaching viral diagnostics

A key innovation of this model is its ability to simulate both sandwich and competitive ELISA formats using magnetic interactions. Traditional teaching of the Hepatitis B Panel often relies on memorization of which markers correspond to which infection stages, without deep understanding of the underlying immunological principles. The model addresses this gap by making the “antigen-antibody binding logic” visible and tangible. Student comments such as “I finally understand how sandwich ELISA works” and spontaneous peer teaching observed during laboratory sessions suggest that the model facilitates genuine conceptual understanding rather than rote memorization.

The model’s integration of viral structure, life cycle, and diagnostic principles into a single cohesive tool is particularly valuable for virology education. Students can observe how structural features (such as HBsAg on the envelope) relate to clinical detection (HBsAg as a diagnostic marker) and therapeutic interventions (neutralizing antibodies blocking HBsAg). This holistic approach mirrors the integrated thinking required in clinical virology practice.

### 4.3 Comparison with existing educational tools

Compared to traditional two-dimensional diagrams, our model offers the advantages of three-dimensional visualization and physical manipulation. While virtual reality simulations can provide immersive experiences, they often require significant technological infrastructure and may not be accessible in all educational settings. Several physical models of viral capsids have been developed for educational and research purposes, each addressing different aspects of virus biology. In the educational domain, many models have focused on virus capsid assembly. Olson et al. demonstrated that 12 identical pentagonal tiles equipped with oriented magnets could self-assemble into a dodecahedral capsid, mimicking the principles of spherical virus self-assembly (Olson et al., 2007). Building on Olson’s design, Höst et al. conducted a controlled study comparing a tangible self-assembling virus model with a static image, revealing that the tangible model better supported students’ understanding of virology (Höst et al., 2013). Their findings provide direct empirical support for the pedagogical value of tactile models in virology education. More recently, Plante et al. developed 3D-printed helical capsid models to illustrate the self-assembly principles of helical viruses, such as the tobacco mosaic virus (Plante et al., 2024). Additionally, Jungck et al. analyzed viral capsid assembly (Jungck et al., 2022; Jungck et al., 2026). While these models offer valuable demonstrations of how capsomers assemble into nucleocapsids, their pedagogical role for students majoring in clinical medicine is relatively limited, as they primarily illustrate the assembly step of the viral life cycle, without integrating other stages such as entry, diagnosis, or therapeutic intervention. Another category of mechanical models focuses on viral capsid expansion. Kovács et al. introduced double-link expandohedra, which are mechanical models in which rigid polyhedral faces are connected by pairs of spherical-jointed bars, to simulate the pH-driven swelling of viruses such as the cowpea chlorotic mottle virus (CCMV) (Kovács et al., 2004). Arstall et al. created both virtual and physical models to visualize the asymmetric expansion pathway of equine rhinitis A virus (Arstall et al., 2015). Dobras et al. designed a 3D mechanical prototype of poliovirus that demonstrates the conformational change from the inactive to the active state, using an iris mechanism to open a pore at the fivefold axis (Dobras et al., 2020). While these models excel in teaching capsid dynamics and genome release mechanisms, they do not address clinical diagnostics or therapeutic interventions. de Souza et al. developed a low-cost microscope slide kit called “Virus Goes Viral,” featuring stained giant virus particles and virus-induced cytopathic effects. While highly accessible and safe, this kit primarily offers 2D visualization rather than a 3D manipulative experience (de Souza et al., 2020). Our model distinguishes itself from these existing tools in three key aspects. First, unlike models that simulate capsid self-assembly or expansion dynamics, our model does not focus solely on nucleocapsid kinetics. Instead, it consists of two magnetic hemispheres that maintain model stability while being openable and transparent, allowing students to physically explore the complete and detailed structure of the hepatitis B virus. This includes the envelope with its three surface antigens, the icosahedral capsid, the partially double-stranded rcDNA genome, and the associated viral polymerase. Second, our model uniquely integrates viral structure with diagnostic principles (including both sandwich and competitive ELISA) and therapeutic mechanisms (e.g., interferon, nucleoside analogs, and neutralizing antibodies) into a single cohesive teaching tool. This feature, which is absent from previously reported physical virus models, bridges structural virology, immunology, and clinical education. Finally, our model specifically focuses on the HBV, a major global health threat for which no comparable tangible teaching model has been previously reported. Our low-cost, tactile model offers a complementary approach, particularly suitable for resource-limited environments or for students who benefit from hands-on learning experiences.

The preliminary evaluation data, although limited in sample size, suggest learning gains comparable to those reported for other hands-on microbiology teaching tools. The improvement from 62% to 84% on knowledge assessment represents a substantial increase, and the qualitative feedback indicates that students found the model engaging and conceptually clarifying.

### 4.4 Broader applications and future directions

The design principles underlying this HBV model are readily transferable to other viruses. We have previously used a similar approach to construct a model of influenza A virus, demonstrating the feasibility of this methodology (Li and Zhang, 2025). The same strategy of using magnetic interactions to simulate antigen-antibody binding and modular components to represent viral structures could be extended to create models of other clinically important viruses, such as HIV, HCV, or SARS-CoV-2. Such a series of virus models could form the foundation of a comprehensive “Modeling viral pathogens” curriculum, allowing students to compare structural features, replication strategies, and diagnostic approaches across different virus families.

This work represents a significant innovation in the functional use of physical teaching tools for viral testing procedures. Unlike the influenza model, which focuses primarily on structural display, the HBV model incorporates an innovative interactive “ELISA simulation kit” that transforms the abstract serological principles of the “Hepatitis B Panel” into tangible, hands-on operations. By providing complete coverage from basic viral structure to clinical diagnostic markers, this model addresses a gap left by previous models in medical education. It effectively helps students build systematic connections between virology knowledge and clinical practice, facilitating a pedagogical transition from fundamental theory to integrated application skills.

This model also has potential applications beyond undergraduate education. It could be used in continuing medical education for healthcare professionals, in public health outreach to improve community understanding of hepatitis testing, or even in patient education to explain diagnostic results and treatment options.

### 4.5 Limitations

This study has several limitations. The educational effectiveness of the model has not been quantitatively assessed through controlled studies comparing it to traditional teaching methods. Additionally, the evaluation is based on a small sample of students from a single institution, which may limit its generalizability to broader populations. The knowledge assessment utilized five multiple-choice questions that were not formally validated, and long-term retention of learning was not measured. Furthermore, the model’s impact on clinical reasoning skills or diagnostic interpretation has not been evaluated. Despite these limitations, the consistency between quantitative gains (22% improvement) and qualitative feedback suggests that the model holds promise as an effective teaching tool, warranting further investigation with larger samples.

### 4.6 Future research directions

Future research will address these limitations through a randomized controlled trial with larger and more diverse student populations. This trial will compare learning outcomes between students using the tangible model and those receiving traditional instruction, measuring both immediate learning gains and long-term retention at multiple time points. We also plan to develop validated assessment instruments that capture not only factual knowledge but also conceptual understanding and clinical reasoning skills.

Additional research directions include investigating the model’s effectiveness in different educational contexts (e.g., medical schools, nursing programs, public health training), exploring its integration with digital learning resources, and developing and evaluating models for other viruses to assess the generalizability of this pedagogical approach.

## 5 Conclusion

This HBV model transforms complex virological concepts into an intuitive and memorable learning experience for undergraduate students. It is easy and cost-effective to create, and students exhibited high engagement during laboratory sessions when working in groups to use the model. Preliminary observations suggest that the model may support student understanding of hepatitis B markers and clinical diagnostics. Formal assessment of learning outcomes is ongoing. As a teaching tool, it effectively addresses key challenges in microbiology education, especially when integrated with multimedia resources. Furthermore, the model holds promise for enhancing public understanding of HBV prevention and treatment, thereby helping to overcome barriers in the current HBV diagnostic and therapeutic processes and contributing to the global strategic goal of eliminating hepatitis epidemics.

## Data Availability

The raw data supporting the conclusions of this article will be made available by the authors, without undue reservation.
